# Phylogeographic history of flat periwinkles, *Littorina fabalis* and *L. obtusata*

**DOI:** 10.1186/s12862-019-1561-6

**Published:** 2020-02-10

**Authors:** Graciela Sotelo, Mårten Duvetorp, Diana Costa, Marina Panova, Kerstin Johannesson, Rui Faria

**Affiliations:** 1 CIBIO, Centro de Investigação em Biodiversidade e Recursos Genéticos, InBIO, Laboratório Associado, Universidade do Porto, Vairão, Portugal; 2 Department of Animal and Plant Sciences, University of Sheffield, Sheffield, S10 2TN UK; 3 Department of Marine Sciences, Tjärnö, University of Gothenburg, Strömstad, Sweden; 4 IBE, Institute of Evolutionary Biology (CSIC-UPF), Department of Experimental and Health Sciences, Pompeu Fabra University, Barcelona, Spain

**Keywords:** Calreticulin, Genetic diversity, Glaciations, Hybridization, Introgression, Marine gastropods, Mitochondrial DNA, Thioredoxin peroxidase 2, Phylogeography, Refugia

## Abstract

**Background:**

The flat periwinkles, *Littorina fabalis* and *L. obtusata*, are two sister species widely distributed throughout the Northern Atlantic shores with high potential to inform us about the process of ecological speciation in the intertidal. However, whether gene flow has occurred during their divergence is still a matter of debate. A comprehensive assessment of the genetic diversity of these species is also lacking and their main glacial refugia and dispersal barriers remain largely unknown. In order to fill these gaps, we sequenced two mitochondrial genes and two nuclear fragments to perform a phylogeographic analysis of flat periwinkles across their distribution range.

**Results:**

We identified two main clades largely composed by species-specific haplotypes corresponding to *L. obtusata* and *L. fabalis*, with moderate to strong support, respectively. Importantly, a model of divergence with gene flow between the two species (from *L. obtusata to L. fabalis*) was better supported, both in Iberia and in northern-central Europe. Three mitochondrial clades were detected within *L. fabalis* and two within *L. obtusata*, with strong divergence between Iberia and the remaining populations. The largest component of the genetic variance within each species was explained by differences between geographic regions associated with these clades. Our data suggests that overall intraspecific genetic diversity is similar between the two flat periwinkle species and that populations from Iberia tend to be less diverse than populations from northern-central Europe.

**Conclusions:**

The phylogeographic analysis of this sister-species pair supports divergence with gene flow. This system thus provides us with the opportunity to study the contribution of gene flow and natural selection during diversification. The distribution of the different clades suggests the existence of glacial refugia in Iberia and northern-central Europe for both species, with a main phylogeographic break between these regions. Although the genetic diversity results are not fully conclusive, the lower diversity observed in Iberia could reflect marginal conditions at the southern limit of their distribution range during the current interglacial period.

## Background

It is now widely recognized that reproductive isolation can progress in the face of gene flow [1]. However, examples where gene flow actually drives speciation (e.g. hybrid speciation or reinforcement) are less common [2]. Phylogeographic studies are an important source of information to understand the evolutionary history of a species [3]. Besides contributing to elucidate the demographic history of populations across the species distribution range, they provide crucial data to infer the geographic context of divergence between populations or species where specific hypotheses, such as the role of gene flow in speciation, can be further tested.

A well-established phylogeographic model for temperate regions of the Northern hemisphere claims that during Pleistocene glaciations many species were able to persist within refugia located at lower latitudes; and when the environmental conditions improved, i.e. during interglacial periods, populations expanded from southern refugia, in some cases meeting and hybridizing at higher latitudes [4–7]. These cyclic events of isolation followed by gene flow after secondary contact are known to have shaped the genetic diversity of many different species distributed across these regions [6, 8] and could even have contributed to speciation [9]. However, this phylogeographic model is probably an oversimplification particularly among marine taxa inhabiting the intertidal realm, as northern refugia during the last glacial maximum (LGM) have been suggested for several marine species, both on the western and eastern sides of the Atlantic [10, 11].

Intertidal species had to face both a reduction of suitable habitats during glacial periods due to ice-sheets covering most northern coastal areas and dramatic changes in the shoreline (sea level) within refugial areas [12–14]. These shifts not only had the potential to change the local selective pressures, in terms of wave exposure etc., but also to foster the contact and gene flow between populations and closely related species. Thus, in order to fully understand the phylogeographic patterns of intertidal species it is important to analyze the genetic diversity across the entire species range, without making assumptions about the location of refugia or the areas where hybridization and gene flow are likely to have happened.

Since the fossil record is often incomplete, the comparison of phylogeographic patterns among closely related species with a similar geographic distribution can be very useful to identify extrinsic barriers to gene flow impacting on both species or to disclose differences related to species-specific life history or ecological requisites [15–19]. For instance, closely related species with similar dispersal capacity and historical distribution range but showing distinct levels of genetic diversity when facing marginal habitat conditions can inform us about the ability of different taxa to resist environmental changes in different regions across their range [20–23]. This is fundamental to accurately predict the species responses to natural or human-mediated climate changes. Comparative phylogeographic studies between sister species can, in addition, reveal where and when introgressive hybridization may have occurred and its role in adaptation and speciation [2, 24].

The flat periwinkles comprise two sister species of the genus *Littorina*, *L. fabalis* and *L. obtusata*, that started to diverge about 1 million years (My) ago (see [25]). They have wide distribution ranges: both are present in the northern Atlantic coasts, from southwest Greenland to Iceland and across the Norwegian Sea to the White Sea; they are present in British Isles and show a rather continuous distribution from Scandinavia to southern Portugal [26]. In addition, *L. obtusata* is also present in the Atlantic coast of North America. The two species have internal fertilization, no free-swimming larvae and a generally low dispersal capacity, although they can occasionally raft longer distances attached to drifting macroalgae, which they also use as their main habitat [26]. Despite the wide range of overlap at the macrogeographic scale, the two species have relevant ecological differences. *L. fabalis* is generally present at the lower intertidal and *L. obtusata* is usually found in the mid-upper intertidal [27]. In non-tidal areas (e.g. Swedish coast) there is instead a division along the shoreline between moderately exposed and sheltered sites: *L. obtusata* is rare or absent in the former while both species are commonly found in the latter [27]. The two species are herbivores, but *L. obtusata* grazes on both *Fucu*s spp. and *Ascophyllum nodosum* (the latter typical from sheltered habitats) whereas *L. fabalis* browses on epiphytes of the former. Predation, mainly exerted by crabs, is thought to have influenced the local distribution and life history of *L. obtusata* and *L. fabalis*, possibly contributing to their divergence [27]. Although the two species are morphologically very similar, adult *L. obtusata* tend to be larger than adult *L. fabalis*, presenting also a longer life-span [28]. There are no shell traits that completely distinguish the two species but male reproductive organs are diagnostic [26, 29, 30].

Gene flow between the two species has been suggested based on sharing of common mtDNA haplotypes throughout the Northeast Atlantic, although incomplete lineage sorting could not be ruled out [25]. In a recent study focussed in the Iberian Peninsula and based on microsatellites, hybrids between the two species were identified in one location in north Portugal [31]. Notably, since the effective population size of mtDNA is usually one quarter of that for nuclear markers, under neutrality, lineage sorting is expected to occur faster in mtDNA than for average nuclear markers [32, 33]. Thus, if shared haplotypes are only observed at mtDNA, introgressive hybridization is supported. Instead, if shared haplotypes are observed in both nuclear and mitochondrial markers, incomplete lineage sorting cannot be excluded. Therefore, the extension of the mtDNA analysis together with nuclear markers along the distribution range is important to clarify how widespread current and past hybridization is in this system.

At the intraspecific level, not much is known about their phylogeographic history apart from the fact that Western Atlantic populations of *L. obtusata* were colonized after the LGM from populations in Europe [34]. For each species, the location of glacial refugia, and whether populations persisted in northern areas during the LGM, as well as of putative phylogeographic barriers have not been previously investigated. Thus, it is currently unknown how population history and genetic diversity have contributed to the vast phenotypic variability observed in this system.

Several ecotypes have been described for *L. fabalis*. In northern Europe, a small (SS) and a large (LM) ecotype are predominantly found in sheltered and moderately-exposed sites in terms of wave action, respectively [35, 36]. In the Iberian Peninsula, three morphotypes/ecotypes are found in exposed (ME), intermediate (FI) and sheltered (ZS) shores dwelling in different macroalgae/seagrass (*Mastocarpus* spp., *Fucus* spp., and *Zostera* spp., respectively) [31, 37]. However, a phylogenetic/phylogeographic analysis of the relationship between these ecotypes is still lacking, and so is an assessment of the relative importance of ecotype evolution and geography in the genetic differentiation between flat periwinkles’ populations.

In this study we address the putative location and impact of glacial refugia, of geographic or ecological barriers to migration, and of hybridization in shaping the genetic diversity of flat periwinkles across their distribution range. To target these questions, we reconstruct the phylogeographic history of the two flat periwinkle species using both mitochondrial and nuclear markers, specifically aiming to understand: i) if introgressive hybridization (i.e. gene flow) occurred during the divergence of the two species; ii) whether northern refugia existed for these species during the LGM; iii) where are the main phylogeographic barriers for each species; iv) if genetic variability differs between *L. fabalis* and *L. obtusata*; and v) if genetic differentiation within *L. fabalis* is more influenced by ecology or geography.

## Results

### DNA sequence variation

**Fig. 1:**
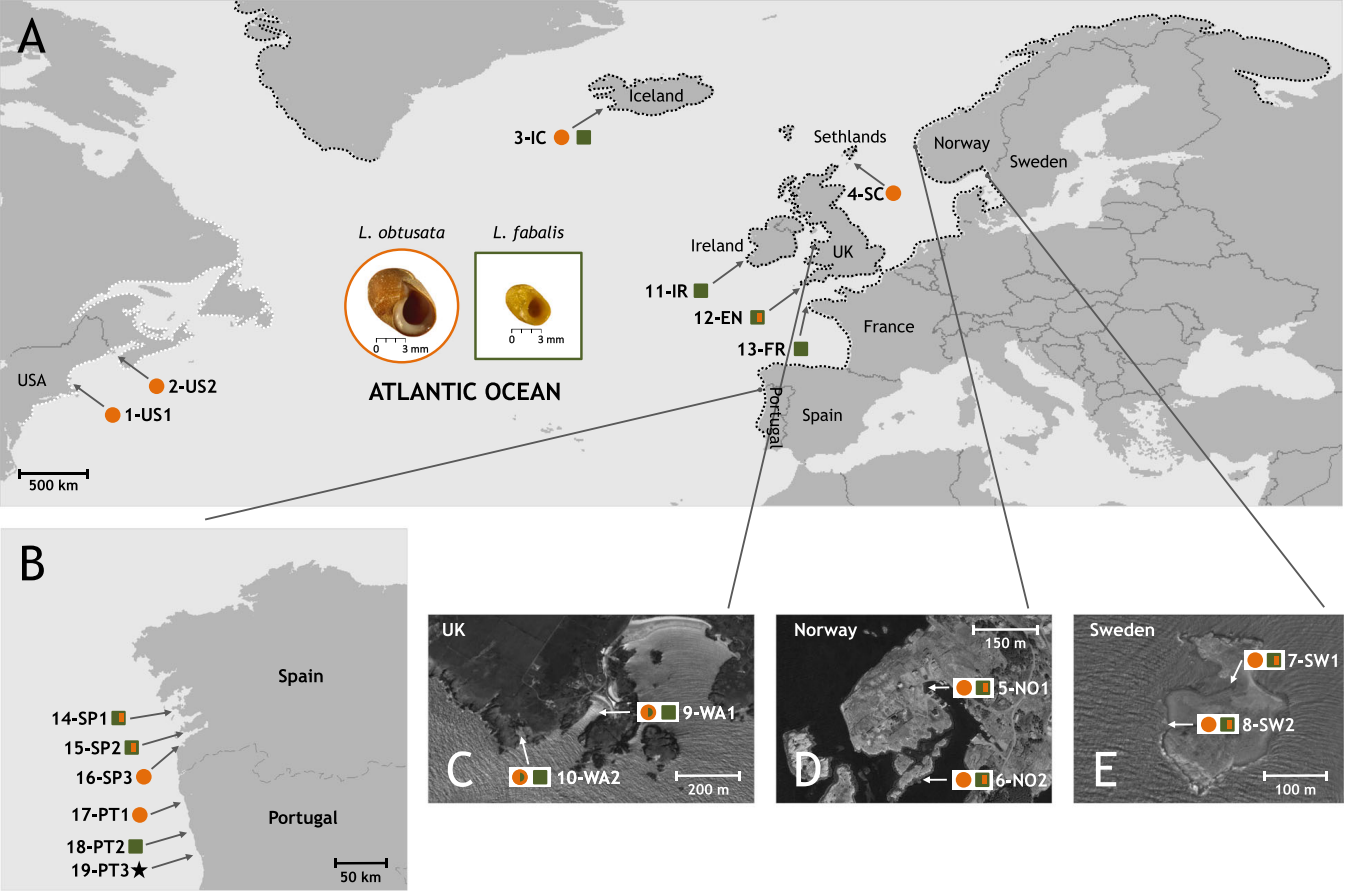
Map of flat periwinkle sampling sites characterized in this study. *Littorina obtusata* samples are represented by orange circles and *L. fabalis* samples by green squares. Symbols filled with both colors (i.e. half orange and half green) indicate populations where mitochondrial introgression was observed: orange circles half filled with green correspond to *L. obtusata* samples carrying typical mtDNA haplotypes from *L. fabalis*; green squares half filled with orange correspond to *L. fabalis* samples carrying typical mtDNA haplotypes from *L. obtusata*; the filling is not proportional to the percentage of introgression. A particular locality where hybrid individuals were previously identified (Cabo do Mundo, Portugal) is represented by a black star. **a** Overview of the sampling sites across the distribution range of the two species, highlighted by dotted shorelines: in white the region where only *L. obtusata* is found and in black the regions where both *L. obtusata* and *L. fabalis* occur along the North Atlantic coast (adapted from [26]). In the middle, *L. obtusata* and *L. fabalis* specimens (from Iberian Peninsula), in the standard position for morphometric analysis of the shell. **b** - **e** Zoom-in of the sampling sites in Iberian Peninsula, Wales, Norway and Sweden. Locality codes follow those in Table 1 with the letters referring to the country. Maps were obtained from DEMIS World Map Server (http://www2.demis.nl/worldmap/mapper.asp) and Google Maps (https://www.google.com) in 2018

The mitochondrial sequence alignments used for phylogenetic inferences were 615 and 585 bp long for *COI* and *Cyt-b*, respectively. These rendered a concatenated mtDNA dataset of 1200 bp sequenced for 344 individuals among the 462 initially available for this study (Table 1, Fig. 1), including 114 different haplotypes (GenBank a.n.: MN045635 - MN045748 and MN045750 - MN045863) defined by 136 variable positions (Additional file 3: Tables S1 and S2). Once the nuclear sequences were phased, the alignment of *Thio* consisted of 511 bp (491 bp after trimming indels as shown in Additional file 3: Table S3), with 19 different alleles (GenBank a.n.: MN061433 - MN061451) found among 225 individuals and defined by 24 variable positions (Additional file 3: Tables S4 and S5); while the alignment of *Cal* consisted of 550 bp (539 bp after trimming indels as shown in Additional file 3: Table S3), with 23 alleles (GenBank a.n.: MN061409 - MN061431) found among 133 individuals and also defined by 24 variable positions (Additional file 3: Tables S6 and S7). Due to their low variability, the nuclear fragments were combined in the nuDNA dataset to infer the interspecific relationships of flat periwinkles. A total of 1022 bp were retained after removing all indels, resulting in 66 different sequences over 102 individuals sequenced for both genes.

**Table 1:** Flat periwinkle samples analyzed in this study. Shown is the locality and country of origin, together with year of collection, geographical coordinates and habitat type. Locality codes include “o” or “f” as prefix when corresponding to *L. obtusata* or *L. fabalis* samples, respectively, followed by the initials of the country where samples were collected (Fig. 1). *N*: sample size (specified by sex: mal, males; fem, females), as the initial number of individuals considered for analysis, followed by the final number of individuals retained in each dataset (*COI + Cyt-b*: concatenated cytochrome oxidase subunit I and cytochrome b; *Thio*: thioredoxin peroxidase 2; *Cal*: calreticulin)

Locality	Country	Year of	Coordinates		Wave Exposure /	Code	*N* Initial	*N COI + Cyt-b*	*N Thio*	*N Cal*
		Collection	Latitude	Longitude	Habitat		(mal/fem)	(mal/fem)	(mal/fem)	(mal/fem)
*L. obtusata*										
Nahant	Massachusetts (USA)	2005	42°25′41.22″N	70°55′43.26″W	NA	(1) oUS1	12 (NA)	10 (NA)	11 (NA)	8 (NA)
Quoddy Head	Maine (USA)	2005	44°48′49.74″N	66°57′3.66″W	NA	(2) oUS2	12 (NA)	8 (NA)	11 (NA)	6 (NA)
Sandgerdi	Iceland	2005	64° 2′0.27″N	22°44′11.82″W	NA	(3) oIC	12 (NA)	10 (NA)	11 (NA)	7 (NA)
Shetland Islands	Scotland (UK)	2007	59°53′6.04″N	1°17′10.71″W	NA	(4) oSC	12 (NA)	9 (NA)	12 (NA)	1 (NA)
Seløyna 1	Norway	2012	60°38′13.62″N	4°47′33.72″E	Sheltered	(5) oNO1	29 (12/17)	15 (9/6)	11 (9/2)	8 (7/1)
Seløyna 2	Norway	2012	60°38′7.08″N	4°47′32.94″E	Exposed	(6) oNO2	4 (2/2)	3 (1/2)	3 (1/2)	3 (1/2)
Lökholmen 1	Sweden	2012	58°53′21.98″N	11° 6′38.61″E	Sheltered	(7) oSW1	12 (9/3)	12 (9/3)	11 (8/3)	10 (7/3)
Lökholmen 2	Sweden	2012	58°53′20.29″N	11° 6′31.94″E	Exposed	(8) oSW2	12 (10/2)	12 (10/2)	12 (10/2)	8 (8/0)
Anglesey South 1	Wales (UK)	2012	53°25′20.30″N	4°22′7.86″W	Sheltered	(9) oWA1	12 (8/4)	12 (8/4)	9 (6/3)	11 (8/3)
Anglesey South 2	Wales (UK)	2012	53°25′5.74″N	4°27′10.82″W	Exposed	(10) oWA2	12 (7/5)	9 (6/3)	11 (7/4)	7 (6/1)
Baiona	Spain	2012	42° 7′26.93″N	8°50′51.35″W	Sheltered	(16) oSP3	40 (28/12)	34 (24/10)	11 (11/0)	11 (11/0)
Rio de Moinhos	Portugal	2012	41°34′4.05″N	8°47′51.81″W	Sheltered	(17) oPT1	40 (30/10)	32 (24/8)	10 (10/0)	4 (4/0)
Total							209	166	123	84
*L. fabalis*										
Sandgerdi	Icleand	2005	64° 2′0.27″N	22°44′11.82″W	NA	(3) fIC	12 (NA)	3 (NA)	5 (NA)	0
Seløyna 1	Norway	2012	60°38′13.62″N	4°47′33.72″E	Sheltered	(5) fNO1	26 (6/20)	12 (4/8)	5 (2/3)	2 (1/1)
Seløyna 2	Norway	2012	60°38′7.08″N	4°47′32.94″E	Exposed	(6) fNO2	12 (7/5)	8 (5/3)	7 (6/1)	5 (3/2)
Lökholmen 1	Sweden	2012	58°53′21.98″N	11° 6′38.61″E	Sheltered	(7) fSW1	12 (12/0)	11 (11/0)	8 (8/0)	8 (8/0)
Lökholmen 2	Sweden	2012	58°53′20.29″N	11° 6′31.94″E	Exposed	(8) fSW2	12 (12/0)	11 (11/0)	8 (8/0)	9 (9/0)
Anglesey South 1	Wales (UK)	2012	53°25′20.30″N	4°22′7.86″W	Sheltered	(9) fWA1	12 (12/0)	9 (9/0)	6 (6/0)	0
Anglesey South 2	Wales (UK)	2012	53°25′5.74″N	4°27′10.82″W	Exposed	(10) fWA2	12 (12/0)	9 (9/0)	5 (5/0)	4 (4/0)
Minard Castle	Ireland	2012	52° 7′12.00″N	10° 6′36.00″W	NA	(11) fIR	12 (12/0)	7 (7/0)	2 (2/0)	3 (3/0)
Wembury	England (UK)	2012	50°18′36.00″N	4° 5′60.00″W	NA	(12) fEN	12 (12/0)	7 (7/0)	2 (2/0)	2 (2/0)
Landunvez	France	2012	48°32′24.00″N	4°44′24.00″W	NA	(13) fFR	12 (12/0)	8 (8/0)	6 (6/0)	0
O Grove	Spain	2012	42°27′45.45″N	8°52′20.59″W	*Zostera*-Sheltered	(14) fSP1	25 (17/8)	21 (14/7)	9 (9/0)	4 (4/0)
Tirán	Spain	2012	42°15′48.01″N	8°45′11.63″W	*Fucus*-Intermediate	(15) fSP2	40 (31/9)	29 (22/7)	5 (4/1)	2 (1/1)
Póvoa de Varzim	Portugal	2012	41°23′7.24″N	8°45′88.30″W	*Mastocarpus*-Exposed	(18) fPT2	24 (12/12)	22 (10/12)	10 (7/3)	3 (3/0)
Total							223	157	78	42
Cabo do Mundo^a^	Portugal	2012	41°13′33.78″N	8°43′3.05″W	Sheltered	(19) PT3	30 (19/11)	21 (12/9)	24 (13/11)	7 (5/2)

^a^Locality where hybrids between *L. fabalis* and *L. obtusata* were previously identified based on microsatellites (see [31])

*NA* not available; Sampling locations code letters stand for the country: *US* USA, *IC* Iceland, *NO* Norway, *SW* Sweden, *WA* Wales, *SP* Spain, *PT* Portugal, *IR* Ireland, *EN* England, *FR* France

### Phylogenetic relationships between flat periwinkles

**Fig. 2:**
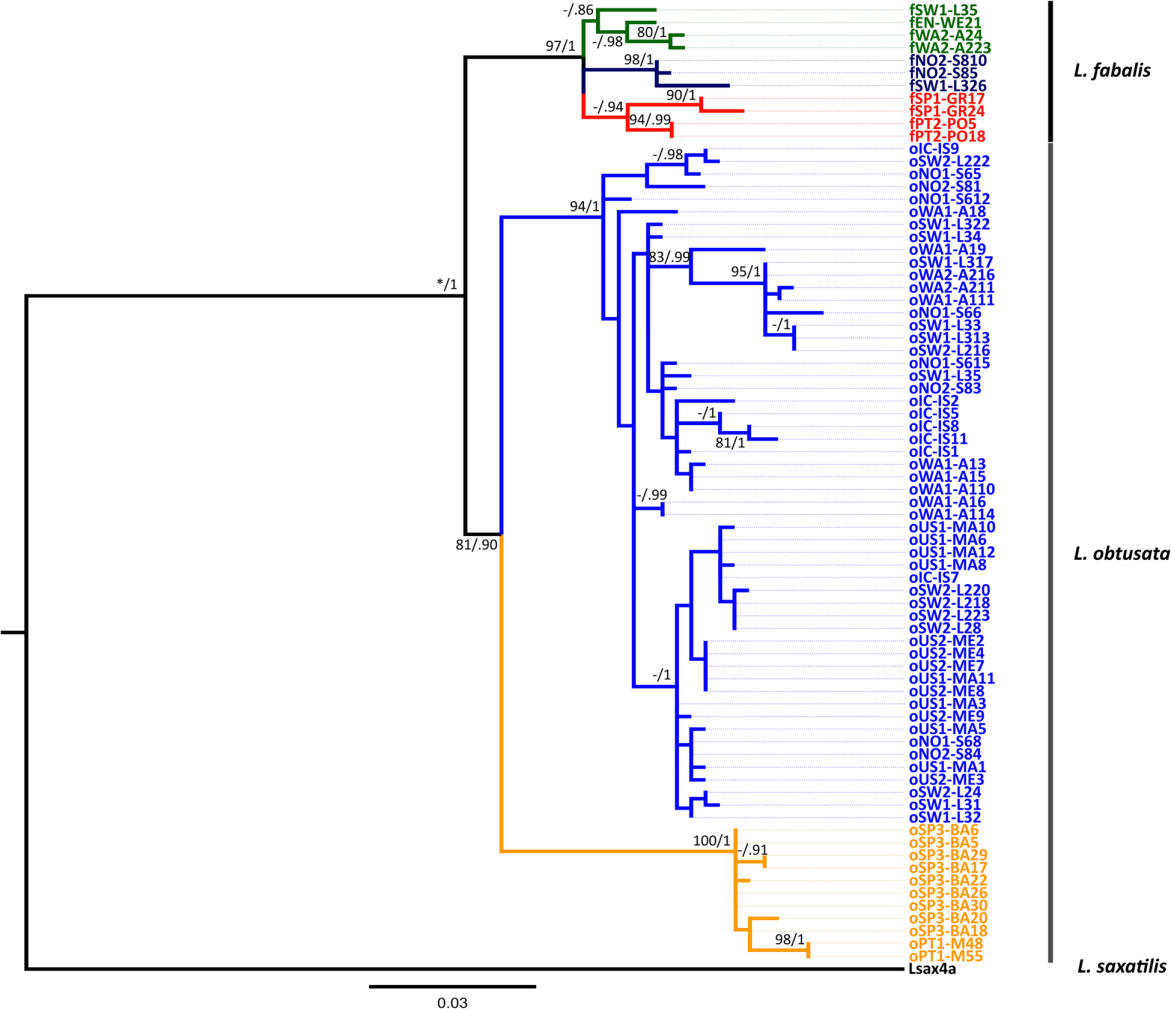
Maximum likelihood (ML) tree for all genes concatenated (mt + nuDNA; 2222 bp). Numbers on branches represent bootstrap values equal to or over 80% for ML (left) and posterior probabilities equal to or over 0.90 for BI (right). (*) Support value not provided by the ML method. The tree was rooted with *L. saxatilis*. Colors refer to lineages, roughly in agreement with geography. For *L. fabalis*, Iceland, Norway, Sweden, Shetlands are indicated in dark blue; UK, Ireland, France, in dark green; and Iberian Peninsula, in dark orange. For *L. obusata*, Iberian Peninsula is indicated in light orange and all remaining samples (USA, Iceland, Norway, Sweden, Shetlands, UK), in light blue

The two flat periwinkles grouped together with a posterior probability of 1 relative to the outgroup for all datasets (mtDNA, nuDNA and mt + nuDNA) (Fig. 2, Additional file 1: Figure S1 and Additional file 2: Figure S2). Two major clades, largely corresponding to the two species, were observed for the mitochondrial and nuclear genes altogether (mt + nuDNA) or the nuclear genes concatenated (nuDNA), but only the monophyly of *L. fabalis* is well supported, while the *L. obtusata* clade presents a moderate support (Fig. 2 and Additional file 2: Figure S2). A similar topology concerning the major clades but with lack of support for the monophyly of each species was obtained for the mtDNA alone (Additional file 1: Figure S1). A reliable divergence time estimate could not be obtained with IMa2 [38] due to lack of convergence. Nevertheless, a rough estimate was calculated assuming a mtDNA divergence rate of 1.76% per My (95% HPD: 1.26–3.14%). This rate was obtained by dividing the average divergence between *L. saxatilis* and flat periwinkles (*Dxy* = 0.044) by the split time between *L. fabalis* and *L. saxatilis* (2.5 My, 95% HPD: 1.4 My-3.5 My [11]). In this way, the pairwise divergence between flat periwinkles (*Dxy* = 0.015) for the mtDNA after removing potential introgressed individuals suggests that the time to the most recent common ancestor (TMRCA) of these two species is around 0.85 My ago (95% HPD: 0.48 My-1.19 My).

### Introgressive hybridization between *flat periwinkles*

**Fig. 3:**
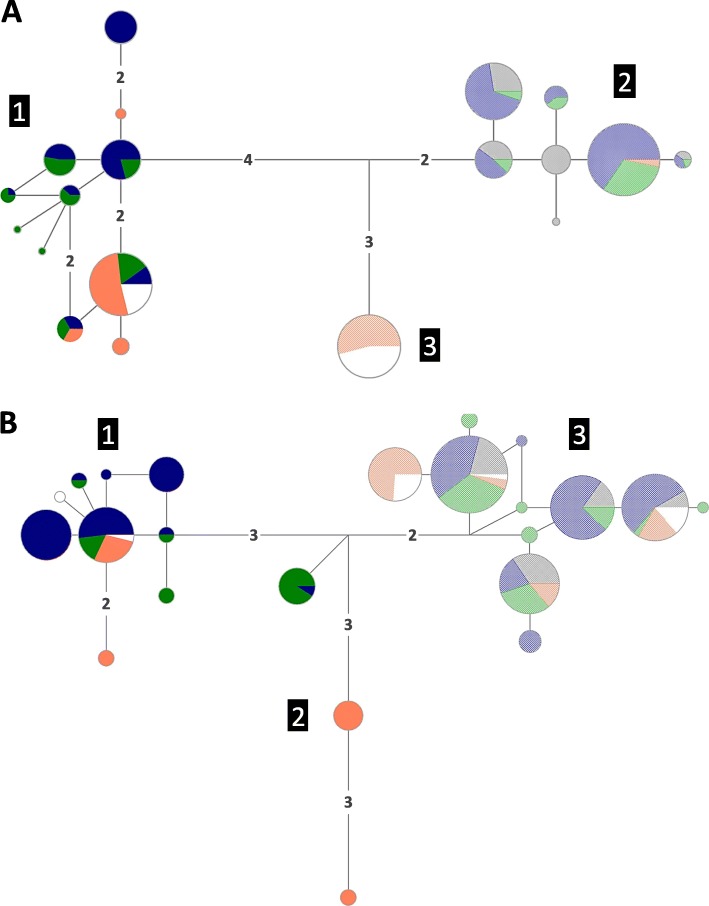
Median-Joining (MJ) haplotype networks for each nuclear gene**. a** *Thio.* **b** *Cal*. Circle areas are proportional to allele/haplotype frequency. Numbers on branches indicate substitution steps between alleles/haplotypes (when more than one). Pie chart fillings correspond to species: solid colored circles – *L. fabalis*; low-transparency colored circles – *L. obtusata*. Individuals from Cabo do Mundo (admixed) are represented in white. The remaining colors refer to lineages, roughly in agreement with geography (North America – grey; Iceland, Norway, Sweden, Shetlands – blue; UK, Ireland, France – green; Iberian Peninsula – orange). Numbers within black squares indicate lineages

The nuDNA data showed that *L. fabalis* and *L. obtusata* do not share any nuclear alleles except for in one of the sampled populations, Cabo do Mundo (Portugal), where hybrids were previously found based on microsatellites [31]. Nine heterozygotes between species-specific alleles were here identified over the 24 individuals from Cabo do Mundo with information for at least one nuclear marker. This supports the former hypothesis of ongoing hybridization in this locality [31] (Fig. 3a and b).

**Fig. 4:**
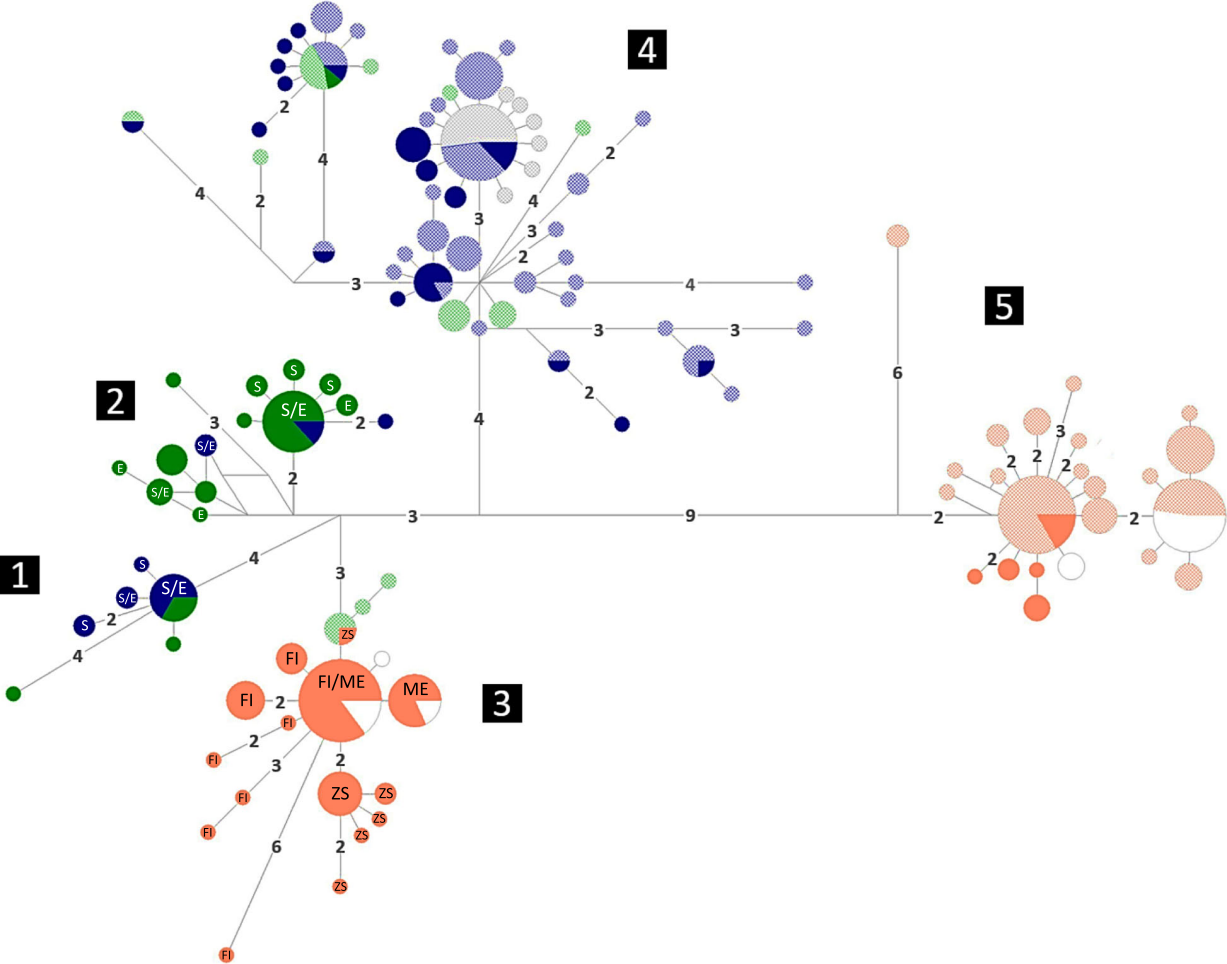
Median-Joining (MJ) haplotype networks for the mtDNA genes concatenated (*COI* and *Cyt-b*). Circle areas are proportional to haplotype frequency. Numbers on branches indicate substitution steps between haplotypes (when more than one). Pie chart fillings correspond to species: solid colored circles – *L. fabalis*; low-transparency colored circles – *L. obtusata*. Individuals from Cabo do Mundo (admixed) are represented in white. The remaining colors refer to lineages, roughly in agreement with geography (North America – grey; Iceland, Norway, Sweden, Shetlands – blue; UK, Ireland, France – green; Iberian Peninsula – orange). Numbers within black squares indicate lineages. Letters within haplotypes refer to *L. fabalis* ecotypes/habitats

In contrast, the mtDNA data revealed a notable amount of haplotype sharing between species (Table 2, Fig. 4). Typical *L. obtusata* haplotypes were found in seven out of 13 *L. fabalis* populations (representing 26% of total *L. fabalis* individuals), with higher incidence in Scandinavia and in particular in the Swedish exposed site (fSW2) where a total replacement of *L. fabalis* haplotypes by those typically found in *L. obtusata* was observed. In contrast, within the 12 *L. obtusata* populations, typical *L. fabalis* haplotypes were only found in the two British populations (oWA1 and oWA2) (representing 3% of total *L. obtusata* individuals). The model-based analysis implemented in IMa2 detected significant migration between the two species (i.e. migration rate significantly different from zero) but only in one direction, from *L. obtusata* into *L. fabalis*. This occurred in the two main geographic regions that were independently analyzed, northern-central Europe and the Iberian Peninsula, while the estimated migration rate was higher in the first (*m* = 0.243 vs *m* = 0.061, respectively).

**Table 2:** Mitochondrial introgression across the flat periwinkle samples included in this study indicated as the number (and percentage) of individuals from one species carrying mitochondrial typical haplotypes from the other species. Codes are the same as in Table 1. Individuals were classified into species using morphology and nuclear markers except for those marked with^a^

Locality	*N COI + Cyt-b*	Introgression
Code	(males/females)	*N* (%)
*L. obtusata*		
(1) oUS1^a^	10 (NA)	
(2) oUS2^a^	8 (NA)	
(3) oIC^a^	10 (NA)	
(4) oSC^a^	9 (NA)	
(5) oNO1	15 (9/6)	
(6) oNO2	3 (1/2)	
(7) oSW1	12 (9/3)	
(8) oSW2	12 (10/2)	
(9) oWA1	12 (8/4)	1 (8%)
(10) oWA2	9 (6/3)	4 (44%)
(16) oSP3	34 (24/10)	
(17) oPT1	32 (24/8)	
Total	166	5 (3%)
*L. fabalis*		
(3) fIC^a^	3 (NA)	
(5) fNO1	12 (4/8)	6 (50%)
(6) fNO2	8 (5/3)	4 (50%)
(7) fSW1	11 (11/0)	8 (73%)
(8) fSW2	11 (11/0)	11 (100%)
(9) fWA1	9 (9/0)	
(10) fWA2	9 (9/0)	
(11) fIR	7 (7/0)	
(12) fEN	7 (7/0)	1 (14%)
(13) fFR	8 (8/0)	
(14) fSP1	21 (14/7)	7 (33%)
(15) fSP2	29 (22/7)	4 (14%)
(18) fPT2	22 (10/12)	
Total	157	41 (26%)

^a^Individuals from these sites were classified into species based on nuclear markers alone

### Phylogeographic patterns, genetic differentiation and diversity

Among the markers analyzed, mtDNA was the most informative at the intraspecific level revealing five main clades that largely correspond to major geographic regions (Fig. 4). Two clades were observed within *L. obtusata*, corresponding to a split between the Iberian Peninsula (clade 5) and the rest of the samples (northern-central Europe and North America – clade 4), with evidence for additional substructure within Iberia due to one very divergent mtDNA haplotype (HM64) present in two individuals from Rio de Moinhos (oPT1), Portugal (Fig. 4). Three clades were observed within *L. fabalis*, essentially corresponding to a split between northern Europe (clade 1) and central Europe (clade 2) apart from the Iberian Peninsula (clade 3). However, some individuals for northern Europe clustered within clade 2 (5 individuals of 39) and some individuals from central Europe within clade 1 (6 individuals of 16, all from France) (Fig. 4). These same clades were also recovered by the mt + nuDNA dataset, although most of them with strong support only under BI (Fig. 2).

The analysis of molecular variance (AMOVA) when populations were grouped by species revealed that most of the variance was explained by differences among species for the nuclear markers (64 and 71%) but it was very similar between species (40%) and among populations within species (43%) for mtDNA (Table 3). The AMOVA within each species separately, when grouping populations by geographic regions taking into account the genetic information described above (two groups for *L. obtusata* and three for *L. fabalis*) showed that most mtDNA variance was explained by differences between geographic regions (73 and 57%, respectively) (Table 4). The mtDNA divergence between the two *L. obtusata* geographic groups was higher than the mean divergence between the three *L. fabalis* groups (*Dxy* = 0.017 vs *Dxy* = 0.007 and *Da* = 0.013 vs *Da* = 0.006, respectively; Table 5), and slightly higher than between the two species (*Dxy* = 0.015 and *Da* = 0.007). Both the AMOVA and divergence estimates are in agreement with an early split between the Iberian clade and the one distributed northwards.

**Table 3:** Analysis of molecular variance (AMOVA, 3-level) including both flat periwinkle species, after grouping populations by species

Structure	Marker	Source of variation	d.f.	Variance components	% Variation	*φ*-statistics	*p*-value
Group1 *L. obtusata* by pop	*Cal*	Among species	1	2.4546	64.22	0.6422	0.0000
		Among populations within species	20	0.4868	12.74	0.3560	0.0000
		Within populations	230	0.8808	23.04	0.7696	0.0000
Group 2 *L. fabalis* by pop	*Thio*	Among species	1	3.2135	70.58	0.7058	0.0000
		Among populations within species	23	0.6908	15.17	0.5157	0.0000
		Within populations	377	0.6488	14.25	0.8575	0.0000
	mtDNA-NOINT	Among species	1	3.5960	39.56	0.3956	0.0000
		Among populations within species	22	3.9936	43.30	0.7165	0.0000
		Within populations	253	1.5576	17.14	0.8286	0.0000

*φ*-statistics presented in the following order: *Φ*_CT_ (up), *Φ*_SC_ (middle) and *Φ*_ST_ (low)

*d.f.* degrees of freedom

*mtDNA–NOINT* mtDNA after removing putatively introgressed individuals

**Table 4:** Analysis of molecular variance (AMOVA, 3-level) after grouping populations by geographic region within *L. fabalis* (upper part) and *L. obtusata* (lower part), based on mtDNA after removing putatively introgressed individuals. fNE1 – *L. fabalis* from northern Europe, fNE2 – *L. fabalis* from central Europe, fIP – *L. fabalis* from Iberian Peninsula. oN – *L. obtusata* from North America and from northern-central Europe, oIP – *L. obtusata* from Iberian Peninsula. Geographic groups were defined according to Fig. 4

Species	Structure	Source of variation	d.f.	Variance components	% Variation	*φ*-statistics	*p-*value
*L. fabalis*	Group 1	Among groups	2	2.4011	56.71	0.5671	0.0010
	fNE1 by pop	Among populations within groups	9	0.7941	18.76	0.4323	0.0000
	Group 2	Within populations	104	1.0386	24.53	0.7547	0.0000
	fNE2 by pop						
	Group 3						
	fIP by pop						
*L. obtusata*	Group 1	Among groups	1	7.5587	73.05	0.7305	0.0137
	oN by pop	Among populations within groups	10	0.8691	8.40	0.3116	0.0000
	Group 2	Within populations	149	1.9198	18.55	0.7305	0.0000
	oIP by pop						

*φ*-statistics presented in the following order: *Φ*_CT_ (up), *Φ*_SC_ (middle) and *Φ*_ST_ (low)

*d.f.* degrees of freedom

**Table 5:** Mitochondrial genetic divergence for the main geographic groups observed within flat periwinkles, after removing potential introgressed individuals

Group	1- fNE1	2- fNE2	3- fIP	4- oN	5- oIP
1- fNE1	**0.0012**	0.0069	0.0081	0.0129	0.0177
2- fNE2	0.0050	**0.0026**	0.0072	0.0121	0.0166
3- fIP	0.0066	0.0050	**0.0018**	0.0130	0.0181
4- oN	0.0095	0.0080	0.0093	**0.0056**	0.0171
5- oIP	0.0158	0.0141	0.0160	0.0131	**0.0025**

Divergence within each group is indicated in the diagonal in bold; *Dxy*, above the diagonal; *Da* below diagonal. fNE1 – *L. fabalis* from northern Europe, fNE2 – *L. fabalis* from central Europe, fIP – *L. fabalis* from Iberian Peninsula. oN – *L. obtusata* from North America and from northern-central Europe, oIP – *L. obtusata* from Iberian Peninsula. Geographic groups were defined according to Fig. 4

Within *L. obtusata,* a lower number of mtDNA haplotypes was observed in the Iberian Peninsula compared with the rest of the distribution of this species (Fig. 4), which rendered significantly lower haplotype and nucleotide diversity in Iberia (oIP) compared with northern-central Europe (oNE) (Tables 6 and 7). The star-like cluster of haplotypes represented by samples from northern-central Europe and North America (oUSA) suggests a recent demographic expansion, which was supported by significantly negative *F*_*S*_ values in both regions as well as significantly negative Tajima’s *D* in North America (Table 6). Haplotype diversity was also significantly lower in Iberia (oIP) than in northern-central Europe (oNE) according to nuclear markers (Additional file 3: Table S8). Similarly, within *L. fabalis* all markers show a higher genetic diversity in northern-central Europe (fNE) than in Iberia (fIP) (Tables 6, 7 and Additional file 3: Table S8), with significant differences in haplotype and nucleotide diversity for the mtDNA and *Thio* data.

**Table 6:** Summary statistics and demographic tests for each population from both species based on the mtDNA dataset (without putatively introgressed individuals). Codes are the same as in Table 1

Species	Code	Habitat	*COI + Cyt-b* (without introgression)						
			*N*	*S*	*h*	*hd*	*π*	*Fs*	*D*
*L. obtusata*			161	91	61	0.9540	0.0106	**−17.0139**	−0.6552
	USA		18	6	7	0.5686	0.0006	**−5.4666**	**−2.0342**
	(1) oUS1	NA	10	4	5	0.6667	0.0007	**−2.8472**	− 1.6671
	(2) oUS2	NA	8	2	3	0.4643	0.0004	−0.9990	− 1.3101
	Northern-central Europe		77	59	37	0.9586	0.0059	**−15.6860**	− 1.3508
	(3) oIC	NA	10	22	8	0.9556	0.0056	−1.2288	−0.6478
	(4) oSC	NA	9	14	6	0.8889	0.0034	−0.4382	−0.9691
	(5) oNO1	Sheltered	15	29	11	0.9048	0.0060	−1.9701	−0.8095
	(6) oNO2	Exposed	3	11	3	1.0000	0.0061	0.8068	0.0000
	(7) oSW1	Sheltered	12	16	6	0.8485	0.0055	1.7877	1.0411
	(8) oSW2	Exposed	12	23	6	0.7576	0.0055	1.7877	−0.6056
	(9) oWA1	Sheltered	11	23	6	0.8364	0.0049	1.1593	−1.1206
	(10) oWA2	Exposed	5	13	2	0.4000	0.0043	4.9368	−1.2104
	Iberian Peninsula		66	31	18	0.8555	0.0026	−5.5098	−1.6721
	(16) oSP3	Sheltered	34	15	10	0.6168	0.0011	−4.8891	−2.1043
	(17) oPT1	Sheltered	32	17	8	0.8085	0.0023	−0.1237	−1.2001
*L. fabalis*			116	50	35	0.9255	0.0053	**−10.1339**	−1.0146
	Northern-central Europe		55	27	20	0.8936	0.0043	−4.2237	−0.4172
	(3) fIC	NA	3	2	2	0.6667	0.0011	1.0609	0.0000
	(5) fNO1	Sheltered	6	2	3	0.6000	0.0006	−0.8584	−1.1320
	(6) fNO2	Exposed	4	9	2	0.8333	0.0038	1.3432	−0.8294
	(7) fSW1	Sheltered	3	10	2	0.6667	0.0056	3.4727	0.0000
	(8) fSW2	Exposed	0	–	–	–	–	–	–
	(9) fWA1	Sheltered	9	8	5	0.8889	0.0019	−0.5590	−1.0265
	(10) fWA2	Exposed	9	6	5	0.8611	0.0025	0.0466	1.5659
	(11) fIR	NA	7	5	3	0.6667	0.0014	1.0142	−0.7926
	(12) fEN	NA	6	7	3	0.6000	0.0019	1.3116	−1.3903
	(13) fFR	NA	8	12	4	0.7857	0.0042	2.1986	0.4043
	Iberian Peninsula		61	25	15	0.8148	0.0018	**−5.5142**	**−1.8661**
	(14) fSP1	*Zostera*-Sheltered	14	8	6	0.6813	0.0011	−2.2485	−1.8766
	(15) fSP2	*Fucus*-Intermediate	25	16	8	0.7800	0.0020	−1.0832	−1.5523
	(18) fPT2	*Mastocarpus*-Exposed	22	1	2	0.5065	0.0004	1.4743	1.4714

*N*, sample size; *S*, number of segregating sites; *h*, number of haplotypes; *hd*, haplotype diversity; *π*, nucleotide diversity; *Fs*, Fu’s Fs; *D*, Tajima’s D

Significant values after Bonferrni correction (*p*-value = 0.025, 0.010, 0.003 for species, regions and populations, respectively) are presented in bold

**Table 7:** Diversity estimates for the major geographic regions for each species, based on mtDNA after excluding putatively introgressed individuals, are indicated in the diagonal (in bold). Haplotype and nucleotide diversity differences between geographic regions are shown below the diagonal, while significance (*p*-values) are shown above the diagonal. o - *L. obtusata*, f - *L. fabalis*, USA – North America, NE – northern-central Europe, IP – Iberian Peninsula

Parameter	Geographic region per species	oUSA	oNE	oIP	fNE	fIP
Haplotype diversity	oUSA	**0.5686**	*0.0000*	*0.0000*	*0.0000*	*0.0000*
	oNE	0.3900	**0.9586**	*0.0000*	*0.0000*	*0.0000*
	oIP	0.2869	−0.1032	**0.8555**	*0.0000*	*0.0010*
	fNE	0.3250	−0.0650	0.0381	**0.8936**	*0.0000*
	fIP	0.2461	−0.1439	−0.0407	−0.0788	**0.8148**
Nucleotide diversity	oUSA	**0.0006**	*0.0000*	0.0270	*0.0000*	0.1800
	oNE	0.0054	**0.0059**	*0.0000*	0.0080	*0.0000*
	oIP	0.0020	−0.0033	**0.0026**	0.0100	0.1870
	fNE	0.0037	−0.0016	0.0017	**0.0043**	*0.0000*
	fIP	0.0013	−0.0041	−0.0008	−0.0024	**0.0018**

Significant values after Bonferrni correction (*p*-value = 0.005) are indicated in italic

Mitochondrial haplotype and nucleotide diversity were, on average, higher in *L. obtusata* than in *L. fabalis*, for each of the two geographic regions where both species are present (northern-central Europe and Iberian Peninsula) (Tables 6 and 7). However, this tendency was not consistent over all localities (Table 6) and only the difference in haplotype diversity remained significant in both regions after Bonferroni correction for multiple testing (Table 7). In contrast, nuclear diversity was in general higher in *L. fabalis* than in *L. obtusata* for each region (NE and IP) (Additional file 3: Table S8), but the difference was only significant in the case of *Thio* for haplotype diversity.

Within *L. fabalis* in the Iberian Peninsula, the population sample of the exposed ecotype (ME, fPT2) had the lowest mtDNA diversity, whereas the population found in *Fucus spp.* (FI, fSP2) had the highest diversity, with the population sample of the ecotype confined to *Zostera* spp. (ZS, fSP1) showing intermediate levels (Table 6). In northern Europe, the number of haplotypes retained after removing putatively introgressed individuals did not allow to reliably compare genetic diversity between sheltered and moderately-exposed sites. Nevertheless, the AMOVA after grouping populations according to wave exposure in northern-central Europe (Norway, Sweden and Wales) revealed no significant differentiation between ecotypes/habitats, while most of the variation was explained by differences among populations within ecotypes/habitats (*Φ*_SC_ = 0.610, *p*-value < 0.001) (Additional file 3: Table S9). In contrast, when populations were grouped according to geography (country), the highest percentage of variation was allocated among countries (57%, *Φ*_CT_ = 0.574, *p*-value = 0.058) (Additional file 3: Table S9).

## Discussion

### Phylogenetic perspective of the diversification of flat periwinkles

The previously proposed monophyly of flat periwinkles [39] was confirmed here. Our results suggest that the TMRCA of the two species is around 0.85 My ago, in agreement with previous estimates based on the entire mitogenome (0.8 My [40]). However, we need to take into account that both estimates are derived from mtDNA data and assume a molecular clock. Different substitution rates and deviations from the neutral or nearly neutral model could render different inferences. Thus, a phylogenomics approach will contribute to improve divergence time estimates between the two species. Interestingly, *L. obtusata* started to diverge into two clades shortly after the divergence between the two species had started. Such relatively close split events could explain the lack of strong support for the monophyly of *L. obtusata*, although the morphology of the reproductive organs (among other traits [41]) supports the existence of two species of flat periwinkles.

### Divergence of flat periwinkles in the face of gene flow

Past mtDNA introgression between the two species was proposed in previous studies [25]. Here we confirm that gene flow has occurred between *L. obtusata* and *L. fabalis* and we also find that it was asymmetric from the former into the latter. This is in line with previous research showing that males from both species prefer large females and mating attempts between small *L. fabalis* males and large *L. obtusata* females are common [42]. If this asymmetry is the result of demographic or selective factors (e.g. adaptive introgression) needs to be further evaluated. However, we cannot exclude that introgression in the opposite direction had also occurred. Three haplotypes present in *L. obtusata* individuals from central Europe clustered with the typical *L. fabalis* haplotypes from Iberia, supporting such introgressive hybridization from *L. fabalis* into *L. obtusata*. This could have happened either in UK, in Iberia before the northward migration, or even during migration as suggested for other species [43]. However, these individuals were not included in the IMa2 analyses as they would violate the model assumptions (see below), and the introgression from *L. fabalis* into *L. obtusata* could not be formally tested using these samples. Intriguingly, no single *L. fabalis* individual carrying typical Iberian haplotypes were observed outside Iberia.

Our results of the isolation with migration (IMa2) analysis also show that introgressive hybridization occurred both in the Iberian Peninsula and in northern-central Europe. Genetic exchange between the two species was higher in the latter region where the two species are more often sympatric, mainly in the sheltered areas of their distribution. However, these results need to be interpreted with caution, since our sampling sites in Iberia are composed mostly from a single species (i.e. allopatric), which could have contributed to a detection of lower introgression when compared with some northern-central European sites.

In contrast, for the nuclear markers, no shared alleles were observed between the two species except in Cabo do Mundo (Portugal) where hybridization had been previously detected [31]. The lack of shared nuclear alleles between species further support the mtDNA introgression scenario, as incomplete lineage sorting is expected if there is more haplotype sharing in nuclear markers on average than in mtDNA, which is the opposite to what we observed. Although the species specific nuclear alleles may be explained by divergent selection (except in Cabo do Mundo), Tajima’s *D* for these markers was not significant in any population or species, as would be expected under a local adaptation scenario. An assessment of the level of gene flow between *L. fabalis* and *L. obtusata* across the genome is a necessary next step to map barrier loci and to understand the extent of the genomic regions involved in reproductive isolation [44, 45].

### Main phylogeographic barriers between populations

Concerning the distribution of intraspecific genetic diversity in this system, the most relevant (and significant) component of the mtDNA variation is due to differences among populations from different geographic regions. The genetic discontinuity between populations from the Iberian Peninsula and those from northern Europe supports the existence of a strong barrier between these regions, which corroborates the patterns observed for other marine taxa (e.g. [14, 19, 46–49] and references therein) including the closely related species *L. saxatilis* [11, 50]. Despite this phylogeographic break, the detection of three haplotypes in five individuals from Wales (UK) clustering into an otherwise Iberian clade suggests that migration, although rare, can have occurred between these biogeographic regions. Future studies aiming to identify putative hybrid zones between Iberian and northern European clades of both species would be useful to inspect the genomic regions involved in possible adaptation to different environmental conditions.

The observation of two clades in northern-central European populations of *L. fabalis* further points to some degree of isolation between UK/France and Scandinavia, which was also detected for *L. saxatilis* [11, 51, 52]. The presence of one divergent haplotype within Iberian populations of *L. obtusata* suggests that clades with deeper divergence exists in the region. Finally, the very shallow divergence between western and eastern Atlantic *L. obtusata* populations is consistent with a recent colonization of the former from northern European populations, probably after the LGM, as earlier proposed [34, 53].

### Comparative genetic diversity and putative location of main glacial refugia

The two species present similar genetic diversity, although it was slightly higher (but significant) in *L. obtusata* than in *L. fabalis* for mtDNA (in terms of haplotype diversity) both in northern Europe and Iberia regions, while the reverse was observed for one of the nuclear markers (*Thio*). Whether these differences reflect a larger female effective population size in *L. obtusata* or simply stochasticity needs to be further investigated, including more nuclear markers and more even sample sizes.

Higher genetic diversity was observed in northern Europe when compared with the Iberian Peninsula both in *L. fabalis* and *L. obtusata*, consistent with the existence of separate refugia in these regions during the LGM. Although such higher diversity in the north could suggest a larger refugium in this region, it is important to emphasize that the larger geographic area covered by our sampling in northern Europe, mainly for *L. fabalis*, in respect to the Iberian Peninsula could have biased our estimates. At the same time, the fact that the Iberian populations are located near the limit of the species distribution ranges [26], where they probably face marginal environmental conditions, could have contributed to higher fluctuations in population sizes and consequently to reduced genetic diversity [54, 55]. The recent disappearance of various fucoid species from the southern limit of their distribution, including the Iberian Peninsula, due to increased temperatures (e.g. [56]; reviewed in [14]) suggests that flat periwinkles are at high risk of a southern contraction in the Iberian Peninsula due to the lack of suitable habitats.

The existence of northern glacial refugia for multiple marine taxa has been previously claimed [10, 57], in particular for the seaweed species inhabited by flat periwinkles ([58, 59]; reviewed in [14]), as well as for the rough periwinkle *L. saxatilis* [11]. Although the exact location of northern refugia for flat periwinkles is yet unknown, since they live and feed on these seaweeds, common refugia for seaweeds and snails seem reasonable.

### Contribution of geography versus ecology to genetic differentiation

Within *L. fabalis*, the differences among the ecotypes/habitats described for northern Europe and UK correspond to a small component of the genetic variation when compared to differences among countries, with populations of each country grouped regardless of the wave-exposure level of the sites where the samples were collected. This suggests that the differentiation between sampling locations independently of the ecotype is more marked than between ecotypes/habitats. Grouping of populations according to geography rather than ecology, at both local or larger scales (within northern Europe and across the entire European shore, respectively) can be interpreted as support for parallel evolution between *L. fabalis* ecotypes. Although a single origin of the ecotypes followed by gene flow within countries can also originate the observed pattern [60], it resembles the genetic variation in mtDNA, nuclear introns and AFLP found in *L. saxatilis* ecotypes across the same geographic regions [51]. However, the future implementation of a demographic modeling approach using multiple markers will be necessary to formally test the hypotheses of parallel divergence of the ecotypes, as in *L. saxatilis* [51]. As well, the identification of markers under divergent selection between ecypes and their distribution across the genome will be crucial to understand the genetic architecture of ecotype divergence in *L. fabalis*.

## Conclusions

Our study supports introgressive hybridization between *L. obtusata* and *L. fabalis* during their divergence. Whether the direction of gene flow is only from the former to the latter, as shown here, or it may also occur in the opposite direction remains an open question. Further studies are needed to assess the role of gene flow in the diversification of flat periwinkles, as well as to identify the genomic regions permeable between species. The distribution of the main genetic clades within each species suggests the existence of a northern refugium for both species besides the one identified in Iberia, as proposed for many intertidal taxa with a similar distribution range. Besides, the high genetic divergence between the Iberian clade and those present at higher latitude suggests that the different ecotypes described for *L. fabalis* in northern-central Europe and those in Iberia could have a different genetic basis. However, this hypothesis requires a proper assessment taking mainly into account the genetic variability involved in adaptive evolution. Finally, despite the different number of populations sampled in each region, the lower genetic diversity observed in Iberian populations, close to the southern distribution limit of both species, compared to northern-central European populations poses some conservation concerns that deserve future attention.

## Methods

### Sample collection

To characterize the phylogeographic patterns of flat periwinkles, samples were collected during 2012 from 19 sites within 15 localities along the northeast Atlantic coast covering most of the distribution range of *L. fabalis* and *L. obtusata* and different degrees of wave exposure (Fig. 1, Table 1). Although the initial aim was to sample sites where the two species do not currently co-exist (i.e. allopatric), this was not possible in some locations of northern and central Europe where neither of the species could be found alone (Fig. 1, Table 1).

To understand the impact of the habitat on the distribution of the genetic variability, representatives of the different ecotypes described for *L. fabalis* [31] were intentionally included. Hence, in northern and central Europe (Norway, Sweden and Wales) samples were collected from both sheltered (5, 7 and 9 in Fig. 1c, d and e) and moderately exposed (6, 8 and 10 in Fig. 1c, d and e) sites, where the SS and LM ecotypes predominate, respectively (Fig. 1c-e). The degree of wave exposure of sampling sites was determined by the abundance of *Ascophyllum nodosum*, following Tatarenkov and Johannesson [36]: this macroalgae is common in sheltered sites but generally absent from moderately-exposed sites. In the Iberian Peninsula (Spain and Portugal), the classification of sampling sites as sheltered, intermediate and exposed was based on the presence/prevalence of different macroalgae/seagrass, following Rolán and Templado [37]: *Zostera* spp., *Fucus* spp. And *Mastocarpus* spp., respectively (sites 14, 15 and 18 in Fig. 1b). However, the relative degree of exposure is only comparable within these regions. Furthermore, for consistency in the classification of sampling sites according to wave exposure within each region, only the sites sharing the same characteristics were classified, avoiding potential differences among locations (Table 1). Finally, samples from the Portuguese locality where hybrids between *L. fabalis* and *L. obtusata* were previously reported (Cabo do Mundo [31]) were also analyzed (site 19, Fig. 1b, Table 1).

Snails were transported alive to the lab, frozen and then transferred to 98–100% ethanol (or directly preserved in ethanol) for posterior analyses. Later on, soft tissues were removed from shells and sexed under a stereomicroscope (Olympus SZx16) with an attached camera (Olympus SDF PLAPO 1XPF). Morphological assignment of individuals to species was done as in Carvalho et al. [31]. Briefly, males were classified based on penis morphology, one of the most used diagnostic characters between *L. fabalis* and *L. obtusata* [26]. According to this criterion, the penis filament is about 30 to 60% of total penis length in the former and 10 to 25% in the latter species (see pages 232 and 206, respectively from [26]). Females were classified based on shell appearance. Since some overlap in shell morphology exists between species [26], whenever possible analyses were preferentially based on males to avoid any potential species misclassification. From all collected individuals, 402 were selected for this study: 211 *L. fabalis*, 161 *L. obtusata* and 30 individuals from Cabo do Mundo (a population mainly composed by admixed individuals, see [31]). In total, males represented 72% of the samples (76, 66 and 61%, respectively) (Table 1).

Sixty samples from four other localities (Iceland, Shetland Islands, Northwest Atlantic coast) that had been previously classified into one of the species (12 *L. fabalis* and 48 *L. obtusata*; Fig. 1, Table 1) were further included. Samples from Iceland and Shetland Islands had been analyzed for a 350 bp fragment of *Cyt-b* by Kemppainen et al. [25]; while samples from Massachusetts and Maine had not been genetically characterized before. All of them were sent as partial tissue samples in ethanol or RNAlater. Importantly, the species classification of these individuals was later confirmed by genetic data (see above). Finally, one sample of *L. saxatilis* was also collected in 2012 from one Portuguese locality (Praia do Carreço) to be used as outgroup.

### Molecular methods

Genomic DNA was extracted from head-foot tissue using either a modified version of the standard high-salt protocol [61], where the lysis buffer was replaced by CTAB buffer [62], or the CTAB – chloroform protocol described in Galindo et al. [63]. DNA quality and quantity were assessed by 1% agarose gel electrophoresis. DNA concentration was standardized across samples by dilution in pure water. Two mitochondrial gene fragments (cytochrome oxidase I – *COI*, and cytochrome b – *Cyt-b*) and two nuclear exon-primed intron-crossing (EPIC) markers (thioredoxin peroxidase 2 – *Thio*, and calreticulin – *Cal*) were amplified using the following primer pairs: LCO1490 – HCO2198 [64], cytbF – cytbR [11], ThioPerF2 – ThioPerR and CalF – CalR2 [51], respectively.

After optimization, for mitochondrial fragments PCR was performed in 25 μL (final volume) containing 1 μL of template DNA, 2.5 μL of 10x reaction buffer, 1 μL of 50 mM MgCl_2_, 1 μL of 10 mM dNTPs (2.5 mM each), 1 μL of 10 μM forward and reverse primers and 0.15 μL of 5 U/μL of BioTaq polymerase (Bioline). PCR cycling conditions consisted of an initial denaturation at 95 °C for 3 min, followed by 35 cycles, each including denaturation at 95 °C for 20 s, annealing from 52 °C to 56 °C for 20 s, and extension at 72 °C for 30 s; and a final extension of 7 min at 72 °C. For nuclear fragments, PCR was performed in 20 μL (final volume) containing 1 μL of template DNA, 2 μL of 10x reaction buffer, 1 μL of 50 mM MgCl_2_, 0.5 μL of 10 mM dNTPs (2.5 mM each), 0.5 μL of 10 μM forward and reverse primers and 0.15 μL of 5 U/μL of BioTaq polymerase (Bioline). PCR cycling conditions consisted of an initial denaturation at 95 °C for 3 min, followed by 35 cycles, each including denaturation at 95 °C for 30 s, annealing from 52 °C to 56 °C for 30 s, and extension at 72 °C for 1 min; and a final extension of 7 min at 72 °C.

PCR products were visualized in 2% agarose gels and purified with Exo I and FastAP (Thermo Scientific). Sanger sequencing was performed at Macrogen Europe (Amsterdam, The Netherlands), using the corresponding primers (forward for mitochondrial and both forward and reverse for nuclear fragments).

### Sequence data and haplotype determination

Chromatograms were visualized and manually checked using Geneious v6.0.5 (http://www.geneious.com), which was also used to align the corrected sequences. No stop codons were observed in the mitochondrial fragments. The two fragments were concatenated in a single dataset (mtDNA) retaining only individuals with available sequence for both genes. Several indels were identified in the nuclear fragments, seven from one to eight bp long in *Thio* and four from one to ten bp long in *Cal* (Additional file 3: Table S3). To assist haplotype reconstruction in individuals carrying alleles with different lengths, a subset of 18 samples (four PCR products for *Thio* and 14 for *Cal*) were cloned using TOPO TA Cloning kit (Invitrogen) according to the manufacturer’s protocol. Cloned fragments were amplified using the universal primers M13F and M13R-pUC and confirmed on agarose gel electrophoresis. After that three to eight clones per sample were sequenced in forward and reverse directions using the M13 primers at Macrogen Europe. Resulting sequences were assembled into contigs in Geneious. For the remaining samples, haplotype phases were manually solved by comparing forward and reverse sequences and using known haplotypes as reference. Indels were reduced to single positions for subsequent analyses (Additional file 3: Table S3). Afterwards, in individuals carrying alleles with the same length but with nucleotide differences at two or more positions, International Union of Pure and Applied Chemistry (IUPAC) ambiguity codes were manually added to those sites. Haplotypes were then inferred with the Bayesian method implemented in PHASE v2.1 [65], incorporating information from all known phases and considering models with (MR0) and without (MS) recombination, while the rest of parameters were set to default. Input files for PHASE were obtained with SeqPHASE [66]. Both models rendered the same result, all phases were unambiguously solved (*p* = 1) except for two individuals from the *Cal* dataset (*p* = 0.5 in one position), which were excluded from subsequent analyses.

Two additional datasets were built by combining the two nuclear fragments alone (nuDNA) and the mitochondrial and nuclear altogether (mt + nuDNA). For the nuDNA dataset, only individuals with sequence available for both nuclear fragments were retained. In this case, indel positions were completely removed from each alignment. Since the phase of the combined haplotypes from the two genes was not known, the two alleles of each locus were also summarized in a consensus sequence assigning back ambiguity codes to heterozygous sites. The mtDNA and nuDNA datasets were subsequently concatenated excluding individuals from Cabo do Mundo (because of their putative hybrid origin) as well as potential introgressed individuals from the remaining localities (i.e. individuals from one species carrying mtDNA haplotypes that clustered within those of the other species) and retaining only individuals with sequence available for both sets. The different alleles/haplotypes in each dataset were identified with FaBox v1.41 [67].

### Phylogenetic inference and phylogeographic analysis

For all datasets (mtDNA, *Thio*, *Cal*, nuDNA and mt + nuDNA), phylogenetic trees were inferred using Maximum Likelihood (ML) and Bayesian Inference (BI) with *L. saxatilis* as outgroup (GenBank a.n.: MN045749, MN045864; MN061452, MN061453; and MN061432), using the best substitution model for each set selected with jModeltest v0.1.1 [68] according to the Bayesian information criterion (TPM3uf + I + G for mtDNA, JC for *Thio*, F81 + G for *Cal*, F81 + I for nuDNA, and HKY + I + G for mt + nuDNA). The ML analyses were done with PhyML v3.1 [69] using the nearest neighbor interchange (NNI) and the subtree pruning and regrafting (SPR) searching algorithms and 1000 bootstrap replicates. Bootstrap values ranging from 80 to 89% were considered moderate clade support, while those equal to or over 90% as strong support. The BI analyses were done with MrBayes v3.2.5 [70], and each consisted of two simultaneous runs starting with random trees, each run with four chains under default heating parameters and 20,000,000 generations sampled every 2000 steps. The standard deviation of split frequencies was used as a convergence index, considering values under 0.01 as indicative of run convergence. The first 25% of the samples were discarded as burn-in and the remaining summarized in a majority rule (50%) consensus tree. Posterior probabilities ranging from 0.90 to 0.94 were considered moderate support, while those equal to or over 0.95 as strong support. ML and BI resulting trees were visualized with FigTree v1.4.0 [71]. For mtDNA, *Thio* and *Cal* datasets, evolutionary relationships between haplotypes/alleles were further depicted by constructing Median-joining (MJ) networks [72] using the software Network 4.6.1.3 (http:// fluxus-engineering.com).

### Divergence between populations and species

Average (*Dxy*) and net (*Da*) divergence between flat periwinkle species as well as between and within intraspecific geographic groups (two for *L. obtusata*: oN – *L. obtusata* from North America and from northern-central Europe, oIP – *L. obtusata* from Iberian Peninsula; and three for *L. fabalis* fNE1 – *L. fabalis* from northern Europe, fNE2 – *L. fabalis* from central Europe, and fIP – *L. fabalis* from Iberian Peninsula; taking into account the phylogeographic information presented in Fig. 4) were further estimated based on *p*-distance from mtDNA data using MEGA X [73]. Divergence (*D*xy) was also calculated between flat periwinkles and *L. saxatilis* (the outgroup), as the average of five different replicates, each consisting of the single *L. saxatilis* individual sequenced here and four randomly chosen flat periwinkles (two from each species, excluding potential introgressed individuals). The aim was to convert nucleotide divergence into time given that the split between *L. fabalis* and *L. saxatilis* has been dated around 2.5 My (95% HPD: 1.4 My-3.5 My) based on partial *Cyt-b* sequences and fossil calibrations [11].

### Isolation with migration model

To test whether the observed sharing of mtDNA haplotypes between *L. fabalis* and *L. obtusata* could be explained to some extent by gene flow during divergence of the two lineages, the isolation with migration model implemented in IMa2 [38] was applied. As the flat periwinkle populations from the Iberian Peninsula were very different from the populations from northern Europe, and as the mtDNA sharing was mainly confined to within regions (see Results), the IMa2 analysis was performed separately for each region. Therefore, a two-populations model was employed in each case: *L. fabalis* vs. *L. obtusata* in the Iberian Peninsula, and *L. fabalis* vs. *L. obtusata* in northern Europe. All individuals from Cabo do Mundo were excluded from the first pairwise comparison because of their hybrid origin. Five *L. obtusata* individuals from Wales were further excluded from the second set because their mtDNA haplotypes clustered with *L. fabalis* haplotypes from the Iberian Peninsula (see Results), violating one of the assumptions of the model: the absence of migration from a third/unknown population. The analysis was based on mtDNA, *Thio* and *Cal* data.

For each analysis, six parameters were estimated: time since population split (*t*), migration rates between current populations (*m1 > 2* and* m2 > 1*), and effective size of current and ancestral populations (*θ1*, *θ2*, *θA*). The HKY model was specified for each locus, and upper prior bounds (splitting time of 10, population size of 25 and 1 for migration) were optimized in preliminary runs. Each final run consisted of 30 chains with geometric heating and 10,000,000 steps after discarding 1000,000 as burn-in. Effective sample sizes (ESS) and trend plots were examined with Tracer v1.6 [74] to assure proper mixing and convergence of each run and consistence across runs. The significance of migration rates (i.e. significantly different from zero) was evaluated through the likelihood ratio test of Nielsen and Wakeley [75].

### Genetic diversity and differentiation between and within species

Hierarchical analyses of molecular variance (3-level AMOVA) were performed to infer the main partition of variance at the species level grouping populations by species, and within each species grouping populations by geographic region but taking also into consideration phylogeographic information (Fig. 4), as described above. Additionally, an AMOVA was performed within *L. fabalis* at the ecotype level, including only sites where two ecotypes/wave-exposure levels were sampled in the same location (i.e. Norway, Sweden and Wales): populations were first grouped by wave-exposure and second by country (regardless of the ecotype), for comparison. The AMOVAs within each species were based on mtDNA data (higher power for intraspecific analyses, see above) after excluding putatively introgressed individuals, while for the species-level AMOVA the nuclear markers were also used.

Genetic diversity was also assessed at various levels: by species, by geographic region within species (North America, northern-central Europe and Iberian Peninsula), and by population (see Table 7 and Additional file 3: Table S8). These analyses were performed for each genetic marker, separately: mtDNA after removing potential introgressed individuals, *Thio* and *Cal*. Molecular diversity was calculated as the number of segregating sites (*S*), number of haplotypes (*h*), haplotype diversity (*hd*) and nucleotide diversity (*π*). Demographic stability was evaluated by means of Fu’s *F*_*S*_ [76] and Tajima’s *D* [77] tests, assuming that the genomic regions analyzed have not been influenced by selection, and their significance was based on 1000 bootstrap replicates and subsequent Bonferroni correction [78] to account for multiple tests. The significance of the difference in haplotype and nucleotide diversity between geographic groups was calculated with the script genetic_diversity_diffs v1.0.3 [79, 80]. All other analyses in this section were performed in Arlequin v3.5 [81].

## Supplementary information

**Additional file 1: Figure S1.** Maximum likelihood (ML) tree for the mitochondrial genes (*COI* and *Cyt-b*) concatenated (mtDNA dataset; 1200 bp). Numbers on branches represent bootstrap values equal to or over 80% for ML (left) and posterior probabilities equal to or over 0.90 for BI (right). The tree was rooted with *L. saxatilis*. Colors refer to lineages, roughly in agreement with geography. For *L. fabalis*, Iceland, Norway, Sweden, Shetlands are indicated in dark blue; UK, Ireland, France, in dark green; and Iberian Peninsula, in dark orange. For *L. obusata*, Iberian Peninsula is indicated in light orange and all remaining samples (USA, Iceland, Norway, Sweden, Shetlands, UK), in light blue. Putatively introgressed haplotypes between flat periwinkles are shown in black.

**Additional file 2: Figure S2.** Maximum likelihood (ML) tree for the nuclear genes (*Thio* and *Cal*) concatenated (nuDNA dataset; 1022 bp). Numbers over branches represent bootstrap values equal to or over 80% for ML (left) and posterior probabilities equal to or over 0.90 for BI (right). The tree was rooted with *L. saxatilis*. Black - *L. fabalis,* grey - *L. obtusata.* (*) Support value not provided by the ML method.

**Additional file 3: **Nine tables (one per sheet) containing information about polymorphic sites and distribution of alleles/haplotypes for the different fragments here analyzed; position of the indels and summary statistics for the nuclear fragments; and AMOVA results for the *L. fabalis* ecotypes.

## Data Availability

Haplotype sequences were deposited in GenBank under the accession numbers MN045635 – MN045864 and MN061409 – MN061453. All other data are available in supplementary material files.

## Abbreviations

°C: Centigrade degree
a.n.: accession numbers
AFLP: Amplified Fragment Length Polymorphism
AMOVA: Analysis of Molecular Variance
BI: Bayesian Inference
bp: base pairs
Cal: Calreticulin
COI: Cytochrome oxidase I
CTAB: cetyl trimethylammonium bromide
Cyt-b: Cytochrome b
DNA: deoxyribonucleic acid
dNTPs: deoxynucleotide solution mix
EPIC: Exon-Primed Intron-Crossing markers
ESS: Effective Sample Sizes
Exo I: Exonuclease I
F1: first generation hybrid
F2: second generation hybrid resulting from the cross between two F1s
FastAP: Thermosensitive Alkaline Phosphatase
FI: *Fucus*-Intermediate - *Littorina fabalis* individuals of the intermediately exposed ecotype from the Iberian Peninsula
fIP: individuals of *L. fabalis* from the Iberian Peninsula
fNE: individuals of *L. fabalis* from northern-central Europe
h: number of haplotypes
hd: haplotype diversity
HPD: Highest Posterior Density
IUPAC: International Union of Pure and Applied Chemistry
LGM: Last Glacial Maximum
LM: Large-Moderately exposed - *L. fabalis* individuals of the moderately exposed ecotype from northern Europe
m: migration rate per generation
ME: *Mastocarpus*-Exposed - *L. fabalis* individuals of the exposed ecotype from the Iberian Peninsula
MgCl_2_: Magnesium chloride
min: minutes
MJ: Median-Joining
ML: Maximum Likelihood
mM: milimolar
mtDNA: mitochondrial DNA
My: million years
NNI: Nearest Neighbor Interchange
nuDNA: nuclear DNA
oIP: indivuduals of *L. obtusata* from the Iberian Peninsula
oNE: individuals of *L. obtusata* from northern-central Europe
oUSA: individuals of *L. obtusata* from the United States of America
PCR: Polymerase Chain Reaction
S: number of segregating sites
s: seconds
SPR: Subtree Pruning and Regrafting
SS: Small-Sheltered - *L. fabalis* individuals of the sheltered ecotype from northern Europe
t: time since population split
Thio: Thioredoxin peroxidase 2
TMRCA: Time to the Most Recent Common Ancestor
U/μL: enzyme units per microliter
UK: United Kingdom
ZS: *Zostera*-Sheltered *- L. fabalis* individuals of sheltered ecotype from the Iberian Peninsula
Θ: effective population size
μL: microliter
π: nucleotide diversity
